# Think outside the box: Incorporating secondary cognitive tasks into return to sport testing after ACL reconstruction

**DOI:** 10.3389/fspor.2022.1089882

**Published:** 2023-02-15

**Authors:** Courtney R. Chaaban, Jeffrey A. Turner, Darin A. Padua

**Affiliations:** Department of Exercise and Sport Science, University of North Carolina at Chapel Hill, Chapel Hill, NC, United States

**Keywords:** functional testing, dual-task, drop vertical jump, single leg hop, cutting, ACL, return to sport (RTS)

## Abstract

The optimal set of return to sport (RTS) tests after anterior cruciate ligament (ACL) injury and ACL reconstruction (ACLR) remains elusive. Many athletes fail to pass current RTS test batteries, fail to RTS, or sustain secondary ACL injuries if they do RTS. The purpose of this review is to summarize current literature regarding functional RTS testing after ACLR and to encourage clinicians to have patients “think” (add a secondary cognitive task) outside the “box” (in reference to the box used during the drop vertical jump task) when performing functional RTS tests. We review important criteria for functional tests in RTS testing, including task-specificity and measurability. Firstly, tests should replicate the sport-specific demands the athlete will encounter when they RTS. Many ACL injuries occur when the athlete is performing a dual cognitive-motor task (e.g., attending to an opponent while performing a cutting maneuver). However, most functional RTS tests do not incorporate a secondary cognitive load. Secondly, tests should be measurable, both through the athlete’s ability to complete the task safely (through biomechanical analyses) and efficiently (through measures of performance). We highlight and critically examine three examples of functional tests that are commonly used for RTS testing: the drop vertical jump, single-leg hop tests, and cutting tasks. We discuss how biomechanics and performance can be measured during these tasks, including the relationship these variables may have with injury. We then discuss how cognitive demands can be added to these tasks, and how these demands influence both biomechanics and performance. Lastly, we provide clinicians with practical recommendations on how to implement secondary cognitive tasks into functional testing and how to assess athletes’ biomechanics and performance.

## The state of play: return to sport after ACL reconstruction

**Figure 1:**
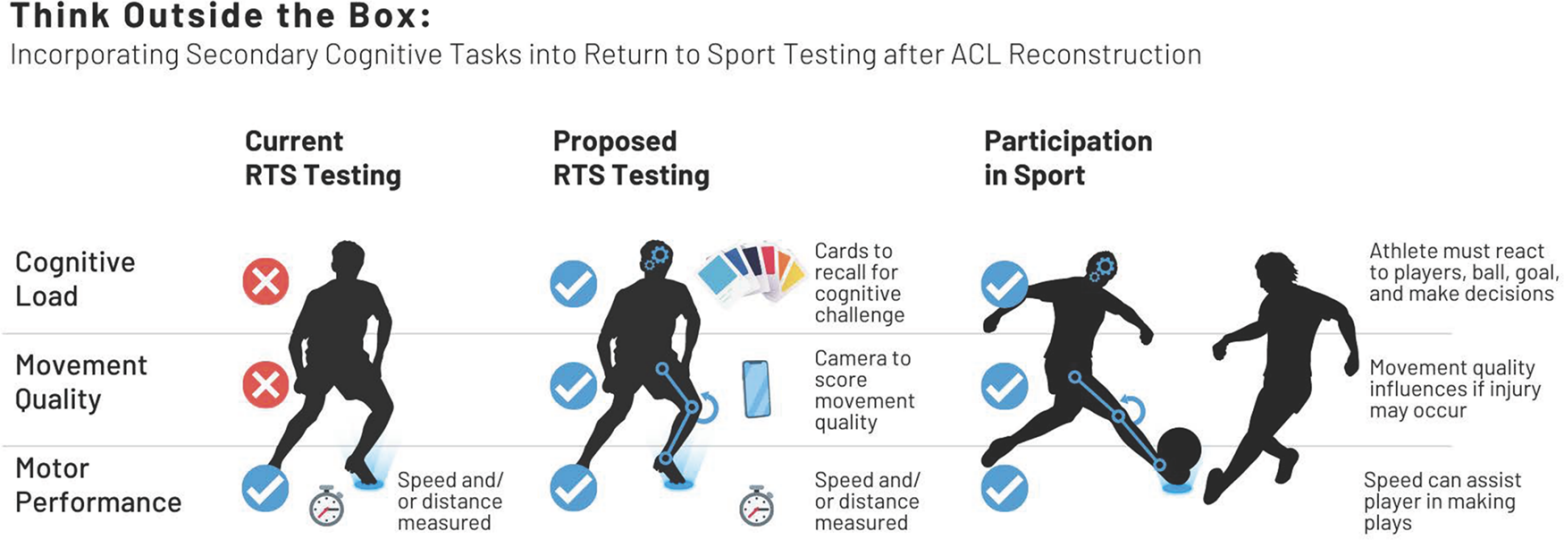
A graphical abstract of current return to sport (RTS) testing, proposed RTS testing, and participant in sport with regard to cognitive load, movement quality, and motor performance.

Following ACL reconstruction, both the ability to return to sport (RTS) and the absence of subsequent ACL injuries are benchmarks for success (1). Unfortunately, many athletes do not meet these benchmarks: approximately one third of athletes do not return to their pre-injury level of sport (2), and up to one third of young athletes sustain a second ACL injury (3–5). These findings lead to critical reflection on current practice, including what modifications can be made to improve outcomes. A graphical abstract of current RTS testing, proposed RTS testing, and participation in sport can be seen in Figure 1, which will be discussed throughout this review.

### The return to sport decision

Patients have high expectations for RTS prior to their ACL reconstruction, with 80%–95% expecting to return to their preinjury level of sport (6, 7). However, reality often fails to meet these expectations, with only 65% returning to their preinjury level of sport (2). The decision-making process regarding RTS is complex and, at times, contentious (8). There are many factors involved in deciding if and when an athlete is appropriate to RTS (9, 10). Likewise, there are many reasons that athletes may not RTS (11). Amidst a challenging landscape with many competing stakeholders (8), RTS test batteries are utilized to assess an athlete’s health status across multiple domains (e.g., signs, symptoms, functional tests, psychological state, etc.) (9).

### Can RTS testing determine who will return to sport?

Historically, time from surgery was one of the only factors used in determining readiness to RTS (12). While time continues to be the most utilized criterion for readiness to RTS, it is often used in conjunction with a battery of tests, which we refer to as RTS testing. RTS testing is utilized for the purpose of informing clinicians in determining who may be appropriate to RTS. Varied RTS test batteries and passing thresholds have been utilized historically (12, 13). Currently, there is still a lack of consensus on the optimal set of criteria or threshold of passing scores to use for a RTS test battery (14). Given that there is heterogeneity across batteries, the ability to draw conclusions regarding RTS test batteries must be tempered. With this in mind, there is evidence to suggest that passing a RTS test battery increases the likelihood of returning to sport (15, 16). However, pooled across studies, there is a low pass rate for RTS test batteries, with between 23% and 43% of patients passing overall (17, 18).

### Can RTS testing determine who will sustain a second ACL injury?

A second ACL injury can occur either by graft failure or by tearing the contralateral ACL. Between 6% and 22% of all individuals after ACL injury will sustain a second ACL injury (19, 20). Both younger age and returning to sport are risk factors of second ACL injury, with a second ACL injury rate as high as 25% to 34% in young athletes who have returned to sport (3–5).

Unfortunately, the relationship between passing a RTS test battery and risk of sustaining a second ACL injury is unclear. Two recent meta-analyses have investigated if passing a standardized, criterion-based RTS test battery influences risk of second ACL injury (17, 18). One meta-analysis found that passing a RTS test battery significantly reduced the risk of graft failure but increased the risk of contralateral ACL injury (18). The other meta-analysis found that passing a RTS battery did not result in a significant reduction in second ACL injury risk (17). Of all studies included in these meta-analyses, only one demonstrated a significant reduction in second ACL injury risk when a RTS battery was passed (21). More recent work has corroborated the idea that passing a current RTS test battery does not reduce the risk of second ACL injury (16). At present, there is not strong enough evidence to support that passing current RTS test batteries, even those with standardized criteria, can discriminate risk of second ACL injury.

### Does RTS testing replicate the scenarios that load the ACL?

In designing RTS test batteries, clinicians should screen for risk of secondary injury through placing athletes in situations that will replicate the demands they will encounter when they participate in sport (22), including those that may make the ACL most susceptible to injury. Video analysis of ACL injuries can provide insight on both the specific task and environment at the time of injury as well as the biomechanics observed. Scenarios that are commonly identified *via* retrospective video analysis of ACL injury include close proximity to opponents, perturbations, deceleration, and cutting (23). While attentional demand cannot be directly observed in ACL injuries, these findings suggest that the ACL is commonly injured when the athlete may be attending to tasks like engaging an opponent or targeting a goal (24, 25). With regard to biomechanics, a consistent finding across video analyses is a relatively extended knee at initial contact with the ground (23, 25–29). Additionally, the ACL tear itself occurs approximately 30–50 milliseconds after initial contact (27, 30), suggesting a very small window of time between initial ground contact and ligamentous failure.

Combining these findings, the ACL may be injured when an athlete has divided attention, hence clinicians should induce similar scenarios during RTS testing. Unfortunately, most RTS tests do not currently incorporate these attentional demands. Additionally, the ACL injury itself frequently includes landing with the knee close to full extension at initial contact. Hence it is important for clinicians to assess biomechanics during RTS testing, including knee flexion angle at initial contact, to identify athletes who land with high-risk biomechanics.

### Summary of the current state of RTS

Passing current RTS test batteries increases the likelihood of returning to sport. However low overall pass rates and an unclear relationship between passing a RTS test battery and secondary injury suggest that there is room for improvement. Additionally, common scenarios of ACL injury are not included in RTS testing. Given the suboptimal outcomes for safe RTS following ACL reconstruction, it is worth examining the apparent disconnect between RTS testing and second ACL injuries after RTS.

Key points:

• Most athletes expect to RTS after ACL injury, but not all do so.
• Athletes who RTS have a high risk of second ACL injury.
• Passing a RTS test battery is not clearly linked to risk of second ACL injury.
• Situations which load the ACL to failure are not incorporated into current RTS testing.

### Keypoints

• Most athletes expect to RTS after ACL injury, but not all do so.
• Athletes who RTS have a high risk of second ACL injury.
• Passing a RTS test battery is not clearly linked to risk of second ACL injury.
• Situations which load the ACL to failure are not incorporated into current RTS testing.
• Current functional RTS tests measure physical performance, such as distance or time.
• Physical performance alone does not provide insight into landing biomechanics and limited insight into injury risk.
• Assessments of movement quality, such as the LESS, that can provide additional information about landing biomechanics outside of a laboratory setting.
• Those with worse cognitive performance demonstrate higher risk biomechanics and elevated ACL injury risk.
• The addition of a cognitive dual-task also leads to higher risk biomechanics and is a common injury scenario in sports.
• Dual-tasks should be incorporated in RTS testing after ACL to inform rehabilitation and readiness to return to sport.
• Cognitive tasks can be added to functional performance tests in clinical settings.
• It is important to assess motor performance and biomechanics.
• Dual-task cost describes how much motor performance and biomechanics change as a result of the cognitive task.

## Current functional performance testing

Functional performance tests are included as a portion of RTS test batteries after ACLR. Some aim to replicate the sport-specific physical demand that individuals may encounter upon RTS. Historically, across 209 studies, functional performance tests have only been used as a portion of RTS test batteries (20% of studies), with hop tests used most frequently (14% of studies) (12). However, this number has increased substantially over time. Since 2010, across 63 studies, 87% of studies utilized the single leg hop for distance test (13), suggesting that functional performance tests are included in the majority of recent RTS test batteries. Despite the inclusion of functional performance tests in RTS test batteries, these test batteries are still controversial in their ability to discriminate who is safe to RTS without sustaining a second ACL injury. Hence, it is reasonable to hypothesize that current functional performance tests may not replicate the demands of sport and/or quantify critical metrics associated with ACL injury.

We will discuss commonly utilized clinic-based functional performance tests, including double-leg drop jump tasks, single-leg hopping tasks, and cutting tasks. While we strongly advocate for the use of additional functional testing in field- and court-based settings, dependent on the specific environment of an athlete’s sport, we elected to focus this discussion on functional performance tests that (1) are easily repeatable in a clinic-based setting, and (2) have clinically feasible assessments of movement quality. We refer to these tests as “functional RTS tests” throughout this manuscript.

### Double leg drop jump tasks

A double leg drop vertical jump task has been used frequently both to screen for primary ACL injury risk as well as a portion of RTS test batteries following ACL injury. When assessing biomechanics utilizing a gold-standard laboratory system, differences have been identified across various timepoints in the ACL injury spectrum. Attempts to predict primary ACL injury risk based on biomechanical variables during this task have yielded conflicting results. One study identified increased knee abduction angle and moment in females who went on to sustain ACL injuries (31). Another study did not find any biomechanical risk factors for primary ACL injury risk (32). Following ACL injury, the ACL-injured limb demonstrates decreased knee extension moment and decreased vertical ground reaction force (33, 34), suggestive of an underloading of the involved knee. Furthermore, those who go on to sustain a second ACL injury also demonstrate sagittal plane knee biomechanical differences relative to those who do not (35–37).

Given that laboratory motion capture systems are not readily available across settings, the Landing Error Scoring System (LESS) task was originally proposed by Padua et al. (38) to assess movement quality during this task. Two standard video cameras provided frontal and sagittal views of the task, and performance was scored based on lower extremity movement quality at two key frames: initial ground contact and maximum knee flexion. Scoring criteria were proposed based on biomechanics associated with risk of non-contact ACL injury, such as knee valgus. Raters reviewed videos to score the LESS. Individuals received a score comprised of the number of errors, with lower scores indicating fewer errors (better movement quality) and higher scores indicating more errors (worse movement quality) (38). The LESS is a valid assessment of lower extremity and trunk biomechanics and has excellent reliability [intraclass correlation coefficient (ICC_2,1_) = 0.91] (38).

Several iterations of the LESS have been identified. A single-camera, markerless system has been developed to automate scoring. This system is reliable compared to expert raters scoring the LESS [Prevalence- and bias-adjusted *κ* (PABAK) = 0.71] (39) and demonstrates moderate agreement against gold-standard markered motion capture to calculate joint angles (40). Notably, the use of the system significantly reduced processing time, which could enhance clinical uptake (39, 41). Another version of the LESS is the LESS-RT (real-time), in which raters score 10 criteria in real-time as an individual performs the same LESS task (42).

There is conflicting evidence regarding whether the LESS has predictive capabilities in prospectively identifying ACL injury risk (43, 44). Following ACL injury, there is also conflicting evidence regarding LESS scores, with one study finding lower LESS scores (fewer errors) in elite female athletes after ACLR compared to their teammates (33), and other studies finding higher LESS scores (more errors) in recreationally active athletes after ACLR compared to matched control athletes (45, 46).

### Single leg hop tasks

The set of four commonly used hop tests (single hop, triple hop, crossover hop, and 6-m timed hop) were first introduced in the early 1990s (47), and they are the most commonly utilized functional RTS test (12, 13). A recent consensus statement found the single hop and crossover hop to have the best measurement properties amongst all functional tests used following ACL injury (48). Additionally, athletes who do not attain symmetrical (>90%) hop distance performance are more likely to sustain ACL graft ruptures (21), suggesting that hop distance symmetry is an important benchmark for a safe RTS.

While these tests have notable strengths, they have also been subject to considerable criticism (49). One criticism of hop testing is that there is no association between performance metrics (distance and time) and biomechanics. Distance on the hop tests is achieved through propulsion during take-off, which is primarily driven by the hip and the ankle (50). In contrast, the knee has a much higher contribution to attenuating landing forces, which is not assessed when looking at distance alone (50). When analyzing symmetry, there is not agreement between distance symmetry and knee kinematics or kinetics (51, 52). Accordingly, a recent systematic review and meta-analysis found that despite the achievement of symmetrical distance, athletes still displayed differences in landing biomechanics post-ACLR, including a decreased peak knee flexion angle and knee flexion moment (53) Athletes post-ACLR compensate on their involved limb during landing through absorbing less work at the knee and more at the hip relative to healthy controls (54).

Combined, these findings suggest that while failure to attain symmetrical distance may have implications for graft rupture, distance alone is insufficient to understand knee loading. Movement strategies used during single leg hop tests appear to be largely independent of performance, suggesting that individuals are able to compensate within the involved limb to achieve a similar performance outcome. Unfortunately, an altered movement strategy may predispose an individual to a secondary injury or abnormal cartilage loading (55). Therefore, incorporating both performance metrics and an assessment of biomechanics concurrently during hop testing would provide a more comprehensive picture.

The single leg hop for distance LESS test (SHD-LESS) has been developed as a qualitative analysis of movement quality during the single leg hop for distance (56). Similar to the original LESS, this test scores movement “errors” at initial contact and maximum knee flexion from both a sagittal and frontal view. This test demonstrated good intra-rater reliability (ICC_2,1 _= 0.87) for scoring in healthy individuals (56). Future research could progress the SHD-LESS similar to the LESS. Automated scoring would significantly improve processing speed and likelihood of adoption. Testing in patient populations would help inform whether or not the SHD-LESS discriminates the ability to RTS and to do so without sustaining a secondary injury. Nonetheless, this type of assessment of movement quality provides additional information not previously captured by traditional measures of performance alone.

### Cutting tasks

Cutting is frequently cited as a mechanism for non-contact ACL injury (25, 27, 29, 57). Sidestep cutting has a knee abduction moment that is six times higher than that of a drop jump (58), suggesting that this could be a more provocative maneuver. While frontal angles appear to have moderate associations between landing and cutting tasks, frontal moments may not be associated (58, 59). Hence, cutting has unique loading patterns that are not captured by jumping and landing tasks. Incorporating cutting as a functional RTS test could provide more specific insight into the movement strategies employed during this high-risk maneuver (60).

When utilizing laboratory-based 3D motion capture and force plates, female soccer players who went on to sustain non-contact ACL injuries during the season demonstrated higher hip adduction angle during a 90-degree cutting task relative to those who did not go on to sustain an ACL injury (61). Following ACL injury, female soccer players who RTS have increased knee abduction angle and internal knee adduction moment compared to those with no history of ACLR (62). These studies suggest that frontal plane loading during cutting tasks differs both prior to and following ACL injury.

The Cutting Movement Assessment Score (CMAS) was developed as a qualitative tool to assess movement quality during a side step cutting task, with scoring on nine variables related to peak knee abduction moment and non-contact ACL injury visual observations (60). The authors suggest using five meters for entry and exit, meaning that some clinics may be constrained in space to execute this test. The CMAS has excellent intra-rater reliability (ICC = 0.95) and moderate-to-excellent inter-rater reliability (ICC = 0.63–0.90) (63). CMAS scores are associated with peak external knee abduction moment as well as greater knee joint loading (63). Similar to findings in laboratory-based settings, CMAS and LESS scores do not associate with each other (64), suggesting that both should be included in RTS batteries as they capture different constructs.

As with the SHD-LESS, additional research is required to understand the CMAS in the context of RTS and secondary injury risk following ACLR. Additionally, as mentioned previously, manual scoring from videos does require significant time. Automation of scoring, similar to the LESS, would significantly improve clinicians’ ability to streamline this test into RTS batteries.

### Current functional RTS testing summary and recommendations

In examining the literature regarding currently utilized functional performance tests, including the double leg drop vertical jump, single leg hops, and cutting tasks, we make the following conclusions:

(1) *Injury prediction*: When utilizing laboratory-based biomechanical assessments, some tasks have modest ability to predict injury risk as well as identify differences after ACLR. However, these differences, if present, are often of small magnitude, requiring laboratory-based assessments to capture them. Additionally, current RTS tasks do not fully replicate the demands of sport. Modifying these tasks to better represent a sports-specific demand may improve their ability to predict injury.
(2) *Motor performance vs. biomechanics*: Current clinical assessments of performance, such as speed or distance, have very limited relationships to biomechanics or injury. Hence, only assessing physical performance could mean missing important information from the test itself that relates to biomechanics that are associated with injury.
(3) *Clinical assessments of biomechanics and movement quality*: While laboratory-based biomechanical testing remains the gold-standard, there are movement quality assessments for each of these functional tests. These assessments score individuals based on the presence or absence of biomechanics associated with injury risk at key video frames. As learned from the LESS, these assessments can be automated with the use of pose-estimation, significantly decreasing processing time.

In critical reflection on current functional RTS testing, we suggest that an ideal functional RTS test should: (1) replicate the sport-specific demands the athlete will encounter, and assess the athlete’s ability to complete the task (2) safely and (3) efficiently. In this definition, “safely” refers to limiting high-risk biomechanical patterns that may strain the ACL to failure. Safe movement quality, which is paramount to the athlete’s continued participation in sport, can be approximated by utilizing a clinical assessment of movement quality, such as the LESS. “Efficiently” refers to the physical performance, including metrics such as speed or distance as applicable. Efficiency is relevant to an athlete’s ability to compete at a high level of sport.

**Figure 2:**
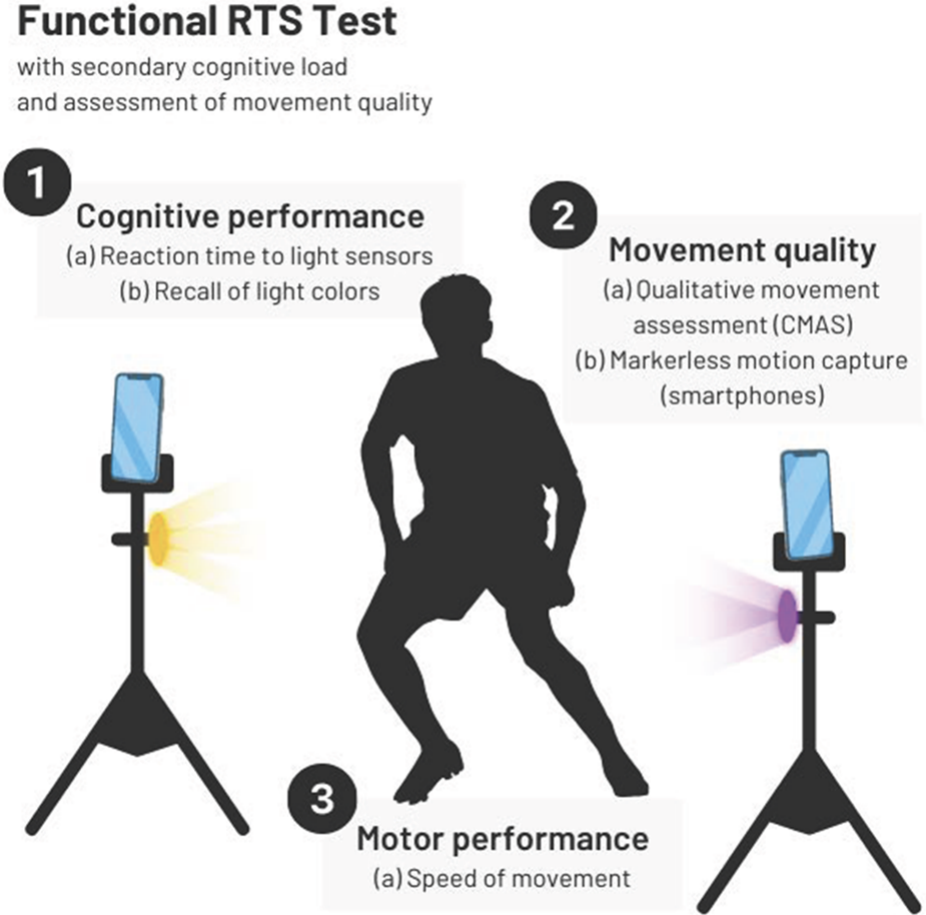
Proposed functional RTS test, including measures of cognitive performance, movement quality, and motor performance. CMAS, cutting movement assessment score.

Current functional RTS tests assess efficiency in specific movements. However, the ability to assess safe movement quality is often constrained to laboratories. Furthermore, most tests are not sports-specific in that they do not incorporate the demands present during sport, including the cognitive loads of attending to other players and tasks. Combining recent recommendations on optimizing RTS, future directions include integration of cognitive stimulation during motor tasks (65, 66). Figure 2 introduces the combined elements of cognitive performance, movement quality, and motor performance that we suggest for functional RTS testing. We will expound upon this introduction in the next section by discussing the addition of cognitive tasks in current RTS tests to better replicate sports-specific demands.

Key points:

• Current functional RTS tests measure physical performance, such as distance or time
• Physical performance alone does not provide insight into landing biomechanics and limited insight into injury risk
• Assessments of movement quality, such as the LESS, that can provide additional information about landing biomechanics outside of a laboratory setting

## Cognitive dual tasks

Individuals in sport must divide attention between stimuli. Often an athlete is required to dual-task (DT): e.g., executing a motor task (jumping, cutting, etc.) while cognitively engaged with a goal (avoiding a defender, reaching a target, etc.). This can be referred to as a cognitive-motor dual-task. Given that athletes returning to sport should demonstrate restoration of both sport-specific function of their injured part and sport-specific skills (67), it follows that these types of dual-task scenarios should be incorporated into functional RTS testing. We will discuss the interplay of cognitive factors with biomechanics and ACL injury risk.

### Cognition’s influence on biomechanics and ACL injury risk

In healthy individuals, baseline differences in cognitive function are related to differences in biomechanics (68, 69). Worse cognitive performance, such as slower reaction time or worse working memory, is associated with higher risk biomechanical loading patterns consistent with ACL injury (68). These differences are seen during both jumping (70) and cutting (71, 72) maneuvers. Differences include higher vertical ground reaction force (vGRF), higher knee abduction angle, higher knee abduction moment, and higher quadriceps activity (68, 72).

Athletes who sustain ACL injuries also have differences in cognitive function measured prior to injury (73). Specifically, college athletes who went on to sustain an ACL injury had significantly slower baseline processing speed and reaction time relative to matched controls who did not go on to sustain an ACL injury (73).

Combined, worse cognitive performance is related to both poor biomechanical movement patterns and risk of ACL injuries. Slower reaction time, processing speed, and working memory suggest that these athletes have constrained ability to process and react to stimuli. Given that ACL injuries happen within 50 milliseconds of initial ground contact (27, 30), these athletes may not have time to implement a safe movement strategy, thereby increasing their risk of injury.

### Cognitive dual tasks’ influence on biomechanics and ACL injury risk

In sport, athletes are often required to divide attention between cognitive and motor tasks. Several studies have demonstrated that adding cognitive tasks when landing or cutting results in higher risk biomechanics loading patterns consistent with ACL injury risk (74–77). A recent narrative review summarized these changes, including reduced knee flexion at initial contact, reduced peak knee flexion, greater peak knee valgus angle, and increased posterior ground reaction force (GRF) (24).

In the case of an individual returning to sport after ACLR, compensatory neural strategies may lead to more difficulty for these athletes in dual-task scenarios, potentially increasing risk of a second ACL injury (78). Therefore, screening for ACL injury risk, including second ACL injury risk following ACLR, should include tasks that incorporate dual-tasks. Doing so would allow clinicians to observe biomechanics present and implement interventions to optimize movement strategies during dual-task scenarios.

Cognitive tasks have been successfully merged with common functional RTS tests. We will summarize current findings and provide direction for future research as well as clinical implementation. We will focus specifically on the addition of a cognitive task to existing functional RTS tests, creating a cognitive-motor dual-task where an individual must divide attention between the two.

### Double leg drop jump tasks

There have been several studies utilizing double leg drop jump tasks in healthy individuals with the addition of a cognitive dual-task. In one such study, the addition of counting backwards (by 1 s and 7 s) resulted in stiffened landings, including decreased knee flexion angle at initial contact and increased vGRF during the first 100 ms of landing (75).

Several studies have also scored the LESS under dual-task conditions. Brazalovich et al. found that in healthy individuals the use of a virtual reality (VR) environment altered landing biomechanics. Specifically, when landing in VR, individuals had increased peak vGRF, less knee flexion at initial contact, and increased knee abduction angle at initial contact (79). These alterations were observed in the VR condition compared to both eyes open and eyes closed conditions, suggesting that manipulation of the environment through the use of VR provided an additional challenge that affected biomechanics. Additionally, worse (higher) LESS scores in the VR condition were observed compared to eyes open or eyes closed conditions, suggesting that the use of the LESS score could similarly detect biomechanical changes in the absence of laboratory-based biomechanical analysis. In another study in healthy individuals, participants performed the LESS task with and without the addition of several cognitive tasks, including the Stroop Color Worst test, Symbol Digits Modalities test, and Brooks Visuospatial task (80). Conversely, this study did not find differences in LESS scores between conditions. However, cognitive scores were worse when in the dual-task conditions compared to baseline cognitive scores, suggesting that these participants may have sacrificed cognitive accuracy for biomechanics.

While some studies have incorporated unanticipated tasks or dual motor-motor tasks to the drop jump task, we are unaware of the use of dual cognitive-motor tasks tested in individuals after ACLR. This represents an area for future research to further elucidate the relationship between performance and biomechanics during these dual-tasks and secondary ACL injury risk.

### Single leg hop tasks

As summarized by Hughes and Dai (24), four studies utilized various single leg landing tasks with the addition of a cognitive dual-task, with three of the four studies demonstrating alterations in biomechanics.

Recent work has incorporated the use of light sensors to provide cognitive dual-tasks during hop tests (81, 82). Using a clinically-feasible set up, physical performance (distance and time) and cognitive performance (reaction time, accuracy) can be measured. These tests are reliable (ICC values all between 0.87 and 0.98) (81) and show decreased performance (distance and time) (82), indicating that the cognitive load condition was sufficiently difficult to induce changes in motor performance. A visual-cognitive medial side hop test has also been developed, which utilizes a visual Space Task (83). This task is reliable [ICC_3,1 _= 0.86 (0.66, 0.94)] for assessment of physical performance (distance) and again demonstrated decreased physical performance compared to the traditional side hop (83).

As with double leg landing tasks, a population for future research is athletes after ACLR.

### Cutting tasks

Cognitive dual-tasks have also been incorporated with cutting maneuvers. One study found that when a serial subtraction task (by 6 s or 7 s) was added to a 45-degree cutting task, healthy individuals demonstrated less peak vGRF and less hip flexion torque (84). Many studies have also introduced decision-making to cutting tasks. This is often done by presenting the individual with a stimulus to indicate the task to perform or direction of the task (e.g., cut to the left vs. the right) (24). These are referred to as unanticipated tasks because the individual does not know the movement they will be performing in advance. Compared to pre-planned tasks, unanticipated cutting tasks commonly result in decreased knee flexion angle at initial contact, increased knee extension moment, and increased knee valgus moment (24). The CMAS has been scored with and without an unanticipated 90-degree cutting task in healthy individuals. The unanticipated task resulted in a higher CMAS score, suggesting more movement errors (85).

As with previous tasks, we are unaware of research examining how the addition of a cognitive dual-task affects athletes after ACLR.

### Summary of cognitive dual tasks

There is a link between worse cognitive performance and both poor biomechanical movement patterns and risk of ACL injury. Likewise, the addition of a cognitive task to jumping, hopping, and cutting tasks alters biomechanics and is commonly the reported mechanism for ACL tears in sports. However, less is known regarding how sustaining an ACL injury, subsequent ACLR, and rehabilitation may further influence an athlete’s ability to complete dual-task scenarios safely and efficiently. Emerging work across double leg landing tasks, single leg hop tasks, and cutting tasks in healthy individuals paves the way for incorporating dual-task scenarios in RTS testing after ACLR.

Key points:

• Those with worse cognitive performance demonstrate higher risk biomechanics and elevated ACL injury risk.
• The addition of a cognitive dual-task also leads to higher risk biomechanics and is a common injury scenario in sports.
• Dual-tasks should be incorporated in RTS testing after ACL to inform rehabilitation and readiness to return to sport.

## Clinical recommendations and future directions

It has been reported that ACL reinjury is as high as 25%–40% following RTS testing and clearance (86, 87). As a clinician, the intent behind RTS testing is to confidently clear an athlete for full return to participation in sport. Current RTS batteries in this population are not meeting expectations and are not comprehensively evaluating athletes. We have outlined that baseline cognitive performance is associated with ACL injury risk (73) and cognitive-motor dual tasks are associated with poor biomechanics during landing and cutting maneuvers (24, 70, 71). Therefore, integrating cognitive performance with functional RTS tasks may serve to optimize efficacy of commonly used RTS test batteries.

While functional performance tests like hop tests do have strengths, they may be enhanced through: (1) understanding the biomechanical strategies used instead of only assessing performance measures, like distance, and (2) incorporating neurocognitive loading to make the tasks better replicate the demands of sports participation. These additions may lead to a test that can better assess readiness to RTS and risk of future ACL injury.

We recommend combining (1) current functional RTS tests that assess motor performance (e.g., the triple hop) with (2) valid and reliable assessments of movement quality (e.g., the LESS) and (3) a cognitive task. As shown above, Figure 2 is a representation of how we envision optimizing functional performance tests during RTS batteries after ACLR. As previous sections of this review have been dedicated to describing the first two, we will discuss how to incorporate cognitive tasks into these RTS batteries.

### Cognitive tasks during functional RTS tests

While many different cognitive tasks have been proposed and utilized for dual-task conditions during training and rehabilitation (88, 89), the requirements for use during functional RTS tests are more constrained. Firstly, the cognitive task must be feasible to perform while completing a motor task. Secondly, the task should have a quantifiable assessment of cognitive performance, such as accuracy or reaction time. Thirdly, the cognitive task should still allow for reliable results in motor performance, movement quality, and cognitive performance such that it could be utilized serially with the same athlete. Additionally, sports often rely on the processing of visual stimuli, hence processing and responding to visual stimuli may be ideal to replicate the nature of sport.

In Table 1, we have proposed ways to incorporate cognitive tasks into RTS testing to satisfy the above criteria. Recognizing that different resources may be available depending on practice location, we have provided both high- and low- technology options, with the goal that these can be adapted to fit available resources without the need for additional expenses. We suggest that these cognitive tasks could be added to any of these motor tasks discussed above, including the drop jump, hop tests, and cutting tasks.

**Table 1:** Suggested cognitive tasks that are measurable and can be added to current functional performance tests.

Category	Task	Resources required	Assessment
Working memory/ recall (series)	Athlete presented with a series of **letters** to view during the task and are asked to recall the letters after. Athlete presented with a series of **colors** to view during the task and are asked to recall the colors after. Athlete presented with a series of **numbers** to view during the task and are asked to recall the numbers after. Athlete presented with a series of **words** to view during the task and are asked to recall the words after.	*Stimulus*: display of series: 1. Printed cards 2. Timed presentation (e.g., Powerpoint) on a computer or tablet 3. Smartphone app 4. Programmed light sensors (e.g., A-Champs ROXPro) 5. Virtual or augmented reality *Assessment*: no resourced required	Accuracy, # of errors
Working memory/ recall (image)	Athlete presented with **one image** of competing stimuli (e.g., various colors for background, text, objects, see Figure 3.) and asked recall a specific component. Athlete presented with **one image** of a sports-specific scenario and asked to recall something about the image after (number of athletes in the image, other details about the image)	*Stimulus:* display of image: 1. Printed image 2. Image on computer or tablet 3. Image projected onto a wall or screen 4. Virtual or augmented reality *Assessment*: no resourced required	Accuracy, # of errors
Anticipation and response inhibition	Visual Go—No Go: athlete presented with **visual stimuli** indicating either to wait or to initiate a movement	*Stimulus*: cards or presentation on computer *Assessment*: timing gate or other type of reaction time/ light sensor	Reaction time, accuracy of movement initiation

**Figure 3:**
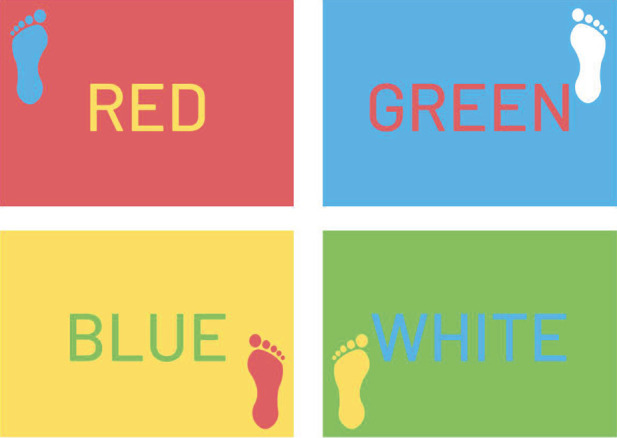
Example cards that could be shown to an athlete during a functional RTS task to add a cognitive load. The athlete could be asked at random to recall the word, the word color, the background color, the footprint color, or the footprint location.

We consider that the barrier to entry to implement these tasks is low. Regarding the presentation of a cognitive stimulus, most clinics have access to computers or tablets that could display a timed PowerPoint presentation with a series of colors, letters, etc. Additionally, there are free smartphone apps [such as SwitchedOn (90)] and free resources online created by rehabilitation professionals (91) to assist in adding a cognitive dual-task. We have adapted a low-technology example (91), which is shown in Figure 3. This figure shows cards that could be presented to the athlete while completing a motor task. Competing stimuli are shown, with conflicting text and colors. The athlete could be asked at random to recall the word, the word color, the background color, the footprint color, or the footprint location. Other resources, such as the light sensors used during the neurocognitive hop tests (81, 82), would require some expense. However, sensors like these are not requisite to implementing many of the tasks proposed in Table 1.

Regarding the assessment of cognitive performance, this is often an assessment of accuracy in responding to the stimulus, which does not require any additional resources. If information like reaction time is to be assessed, timing gates at various price points can be utilized.

Most importantly, the selected task should be one that can be implemented repeatedly. It may be used multiple times within the same session, such as during different tasks or when testing the involved vs. the uninvolved limb. Additionally, it may be used on different days, such as repeating a RTS test battery after several weeks or months.

### Assessment of movement quality

As established previously, the addition of a cognitive task results in decreased physical performance and higher risk biomechanics. If the goal of a RTS test battery is to assess an individual’s readiness to RTS safely, it is paramount to include an assessment of movement quality during the dual-task scenario, as neither motor performance nor cognitive performance alone are sufficient to understand the position and load across the knee.

In most clinics, clinicians can use two cameras in order to score the movement quality assessments discussed previously (LESS, SHD-LESS, and CMAS). We have provided spreadsheets (Supplementary) that explain all errors and scoring for each of these assessments along with a summation to the final score for each assessment.

If clinicians would like additional detail on kinematics, they can look to emerging open-access pose-estimation applications, such as OpenCap (92). This free application relies on two iOS devices to record video and automatically computes three-dimensional kinematics. At this time, clinicians can visualize kinematics within the application. We anticipate that future work will lead to automated scoring of these assessments, similar to what has been done previously with the LESS (39).

### Dual-task cost

According to the capacity model for attention, an individual has a limited capacity for attention at any given time point (93). If an individual is engaged in a dual-task, attention devoted towards one task affects the efficacy of the other (94). This can be referred to as the dual-task cost, where dual-tasking affects performance (95) on one or both tasks. This paradigm is present in the aforementioned studies, where the addition of a cognitive task may affect the motor skill (motor performance or biomechanics) and/or the cognitive skill. A calculation of the dual-task cost, which is the difference in measures with and without the addition of the dual-task, shows the incremental changes as a result of the dual-task scenario. Prior research on the addition of a cognitive task to a medial side hop utilized this calculation (83). We suggest that this is important information to contextualize findings from functional RTS tests with and without a cognitive task.

To illustrate the calculation of the dual-task cost, we present an example in Table 2. In this example, the single leg hop for distance is performed with and without a cognitive challenge. To calculate the motor performance dual-task cost, you can subtract the score in the cognitive condition from the score in the regular condition. Calculating the movement quality dual-task costs works in reverse since a higher score on the SHD-LESS indicates more errors. In this case, you can subtract the score on the regular condition from the score on the cognitive condition. For both cases, in order to normalize the score, you will divide the difference by the regular condition and multiply by 100.

**Table 2:** Example calculation of dual-task cost of motor performance and movement quality.

Task	Motor Performance	Movement Quality	Cognitive Performance
SL hop for distance	Distance regular (100 cm)	SHD-LESS regular (4 errors)	N/A
SL hop for distance + working memory task (recalling 3 colors in order)	Distance cognitive (80 cm)	SHD-LESS cognitive (6 errors)	Recall accuracy (2/3 correct)
Dual-task cost	Distance jumped regular—distance jumped cognitive [(100 cm–80 cm)/100 cm × 100 = 20% cost]	SHD-LESS cognitive—SHD-LESS regular [(6 errors–4 errors)/4 errors × 100 = 50% cost]	N/A

Example values are provided in paratheses.

SL, single leg; SHD-LESS, single hop for distance landing error scoring system.

Prior research suggests there is a dual-task cost associated with both motor performance and movement quality in healthy individuals when a cognitive task is added to a functional RTS test. There are currently no standards established for acceptable performance with the addition of a cognitive load or an acceptable dual-task cost. However, we suggest that there is still clinical relevance in these numbers. If an individual has poor motor performance and/or movement quality scores without the addition of a cognitive task, they likely require additional training on the impairments related to these scores, such as improved quadriceps strength or neuromuscular control. However, if scores without the addition of a cognitive task are acceptable, yet the individual has a high dual-task cost with considerable changes in motor performance and/or movement quality with the addition of a cognitive load, the athlete may benefit from additional training under dual-task conditions.

### RTS decision making

There is not currently empirical data to guide specific cut scores for motor performance, movement quality, cognitive performance, or associated dual-task cost of the proposed tasks. It is unknown if athletes with a higher dual-task cost are more likely to sustain injury. Likewise, it is unknown if ACL injury itself increases dual-task cost. We envision that future research in these areas will help to establish standards as a portion of the RTS test battery.

Until guidelines are established, we advise clinicians to incorporate their clinical decision-making to interpret findings at this time. While traditionally, cut scores such as 90% limb symmetry index (LSI) have been used (12), these may or may not be appropriate in the cases proposed here. Instead, we urge caution in athletes who have large dual-task costs, as this suggests significant decrement in performance when placed in a more sports-specific scenario as opposed to a traditionally assessed task. While these athletes may appear ready to RTS based on a traditional test battery, they may present with high-risk biomechanics during sports-specific training. These athletes would likely benefit from additional intervention to mitigate their injury risk prior to full RTS.

### Clinical implementation summary

There are many options to implement cognitive tasks into functional RTS tests. The options selected should be quantifiable and reliable. It is important to assess an athlete’s biomechanics during a dual-task condition. Clinical movement quality assessments, like the LESS, can be utilized to do so in the absence of other motion capture. Additionally, the calculation of dual-task cost will help clinicians to understand how the addition of a cognitive task alters both motor performance and movement quality, which will help to inform both rehabilitation interventions and readiness to RTS.

**Figure 4:**
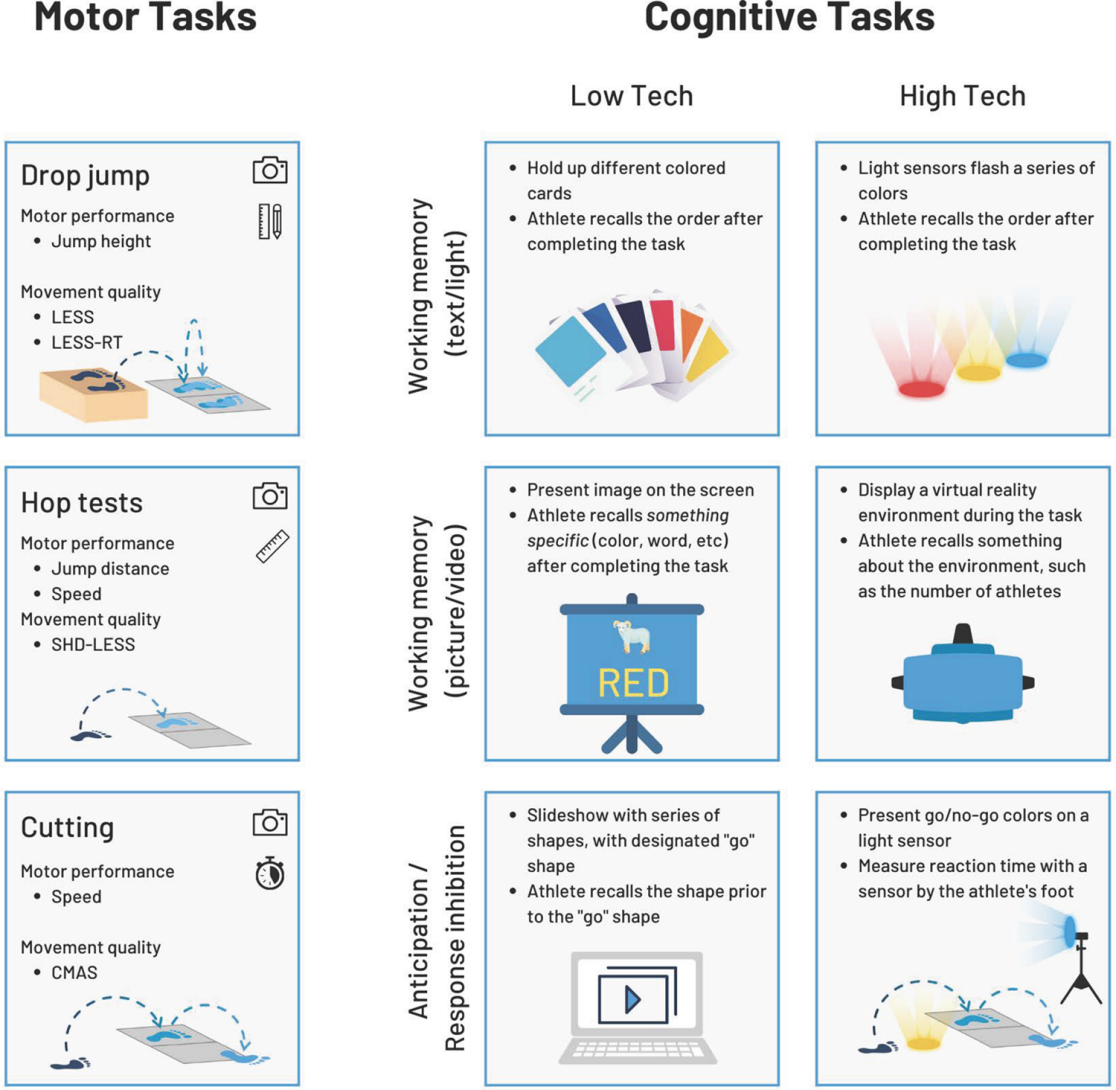
Cards with suggestions on implementation of motor tasks and cognitive tasks. LESS, landing error scoring system. LESS-RT, landing error scoring system real time. SHD-LESS, single hop for distance landing error scoring system. CMAS, cutting movement assessment score.

To support clinical implementation, Figure 4 displays options for both motor tasks and cognitive tasks. Motor tasks include the double leg drop jump, single leg hop tests, and cutting. For each, we list elements of motor performance that can be assessed along with movement quality assessments that can be used. The icons on the top right of each box indicate the resources required. Cognitive tasks are displayed on the right of the figure, including several low and high technology options. Clinicians can mix-and-match one motor task card along with any cognitive task card of their choosing based on resources available in their clinic.

Key points:

• Cognitive tasks can be added to functional performance tests in clinical settings
• It is important to assess motor performance and biomechanics
• Dual-task cost describes how much motor performance and biomechanics change as a result of the cognitive task.

## Conclusions

Current RTS test batteries following ACLR are limited in their ability to determine who is safe to RTS. ACL injuries often occur when an athlete is engaged in some type of cognitive challenge during sport, yet this cognitive load is not present during functional tests in RTS test batteries. Clinicians can “think outside the box” and add measurable, reliable cognitive tasks to these tests. Furthermore, clinicians should quantify both motor performance and movement quality with and without this cognitive task. The dual-task cost is the difference in these measures as a result of the cognitive task. This paradigm can help inform both rehabilitation interventions as well as readiness to RTS.
